# Crosstalk between ferroptosis and NLRP3, a possible therapeutic target in experimentally-induced rheumatoid arthritis: role of P2Y12R inhibition in modulating P53/SLC7A11/ALOX15 signaling

**DOI:** 10.1007/s10787-025-01841-8

**Published:** 2025-07-07

**Authors:** Fatma S. Eltyar, Dalia M. El-Tanbouly, Hala F. Zaki, Rehab M. El-Sayed

**Affiliations:** 1 Department of Pharmacology and Toxicology, Faculty of Pharmacy, Sinai University–Arish Branch, Arish, 45511 Egypt; 2 Department of Pharmacology and Toxicology, Faculty of Pharmacy, Cairo University, Kasr El-Aini St., Cairo, 11562 Egypt

**Keywords:** Rheumatoid arthritis, Ticagrelor, Ferroptosis, SLC7A11, ALOX15, NLRP3

## Abstract

Ferroptosis is critical in progressing and exacerbating rheumatoid arthritis (RA) and other inflammatory joint diseases. Inhibition of the P2Y12 receptors reduced iron overload in macrophages displaying an anti-inflammatory response. Herein, the ameliorative effect of ticagrelor, a reversible P2Y12 inhibitor, against adjuvant-induced arthritis (AIA) in rats was investigated, with a special emphasis on the possible modulation of some inflammatory signals linked to ferroptosis. Particularly, correlation analyses were conducted between nod-like receptor protein 3 (NLRP3) and all assessed parameters. Four groups of rats were assigned: Control group, AIA group (0.1 ml intradermal injection of complete Freund's adjuvant), Ticagrelor group (30 mg/kg, p.o.), and Ticagrelor + AIA group. Ticagrelor exhibited an anti-arthritic effect, evidenced by significant improvements in both macroscopic and histopathological alterations. It effectively inhibited ferroptosis, indicated by a marked upregulation of the ferroptotic inhibitors, solute carrier family 7 member 11 (SLC7A11), glutathione peroxidase 4 (GPX4) and ferritin heavy chain 1 (FTH1) to reach 9.80, 2.20, and 8.49-folds (p < 0.0001), along with a notable reduction in the ferroptotic promoters, P53, acyl-CoA synthetase long-chain family member 4 (ACSL4) and arachidonic acid 15-lipoxygenase-1 (ALOX15) by 89.46%, 41.45% and 49.85% (p < 0.0001). It reduced TNF-α and various chemokines (RANTES, MIP-1α, eotaxin-3) to suppress matrix metalloproteinases expression. Furthermore, ticagrelor decreased NLRP3 expression by 48.63% (p < 0.0001) to pinpoint its anti-inflammatory effect. Overall, amending the P53/SLC7A11/ALOX15 axis by ticagrelor mediated its anti-inflammatory and anti-ferroptotic effects. These findings provide preliminary experimental evidences for further investigating the potential impacts of ticagrelor as a treatment for RA.

**Graphical Abstract:**

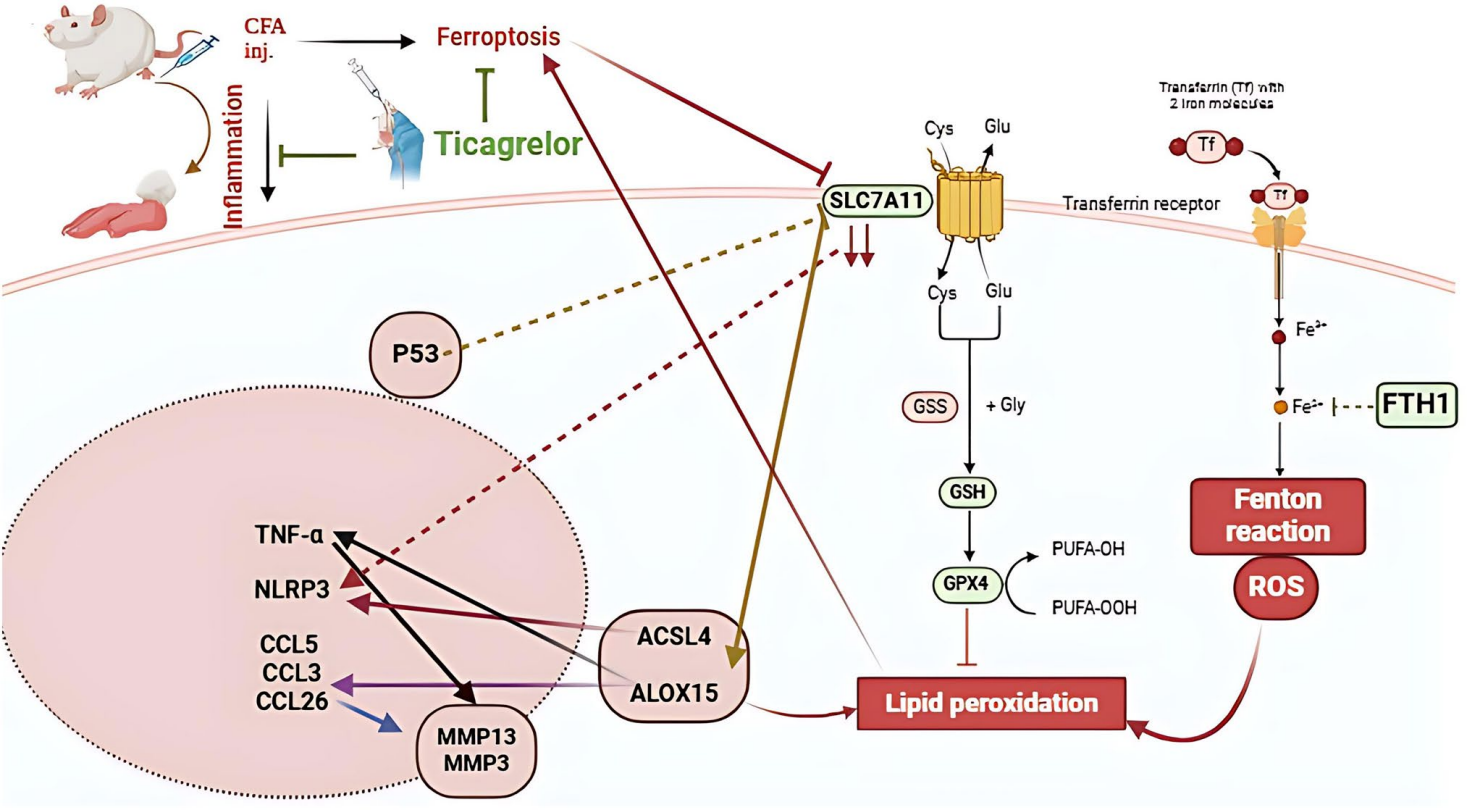

## Introduction

Rheumatoid arthritis (RA) is a systemic autoimmune disorder that refers to the aberrant growth of the synovium, which causes inflammation. This excessive growth invades the cartilage and bone, resulting in the progressive deterioration of the joints (McInnes and Schett 2011; Radu and Bungau 2021). RA affects around 3 in every 10^4^ people around the globe and ranks 42^nd^ highest disability rate among 291 diseases (Nerurkar et al. 2019; Prasad et al. 2023). Due to the obscure pathogenesis of RA and its associated patient suffering, there is an urgent desire for innovative research to develop effective treatments.

Ferroptosis is a newly discovered mode of programmed cell death that is triggered by an excessive iron deposition within cells. Intracellular iron buildup, oxidative degradation of lipids, or perturbations in the system of antioxidants are the primary triggers of ferroptosis, resulting in membrane integrity loss and eventual cell death (Li et al. 2024). Noteworthy, following the entry of ferrous ions into the cytoplasm, a portion of it attaches to ferritin heavy chain 1(FTH1) and undergoes oxidation to produce ferric, which subsequently attaches to ferritin light chain (FTL), forming the ferritin complex, which is then retained within the cell. Excess ferrous ions build up in cells and create unstable iron pools once iron-binding complexes are saturated (Wang et al. 2021). Through the Fenton reaction, excessive ferrous ions can induce substantial reactive oxygen species (ROS) generation, altering the equilibrium of redox processes inside cells, amplifying oxidative damage to lipids, and prompting ferroptosis (Hirayama et al. 2017).

Ferroptosis can only be induced by peroxidase polyunsaturated fatty acids (PUFA) that are integrated into lipids such as phospholipids (Dixon et al. 2012). Acyl-CoA synthetase long-chain family member 4 (ACSL4) is essential for the biosynthesis of membrane phospholipids that contain phosphatidylethanolamines-PUFAs (PUFA-PEs). PUFA-PE is extremely vulnerable to oxidation by lipoxygenases such as arachidonic acid 15-lipoxygenase-1 (ALOX15) and extreme ROS, ultimately resulting in ferroptosis (Li et al. 2024). Furthermore, it can be induced by the impairment of the body's antioxidant defenses (Riegman et al. 2020). The selenium protein, glutathione peroxidase 4 (GPX4) efficiently lowers peroxides and prevents the triggering formation of peroxidized phospholipid throughout the metabolism of arachidonic acid (Friedmann Angeli et al. 2019). Additionally, solute carrier family 7 member 11 (SLC7A11) is the primary subunit of system Xc-, a cystine carrier protein that facilitates the transfer of cystine and glutamate across the cell membranes, promoting the biosynthesis of reduced glutathione (GSH). Thus, disruption of the human body's vital antioxidant system, SLC7A11/GSH/GPX4 accelerates lipid peroxidation, thereby inducing ferroptosis (Chen et al. 2021).

Emerging evidences revealed that ferroptosis promotes the progression and exacerbation of RA and various inflammatory joint disorders. The pathogenic features of RA align with the features of ferroptosis, specifically lipid peroxidation, iron deposition, glutathione loss and GPX4 inactivation (Stockwell 2022; Xie et al. 2021). A growing body of research has reported that the activation of signaling pathways involved in inflammation is very closely associated with ferroptosis. In RA, the alteration of the synovial membrane leads to liberating inflammatory cytokines like tumor necrotic factor alpha (TNF-α) (Telfer and Brock 2004) that induces chondrocytes to release matrix metalloproteinases (MMPs) like MMP13 and MMP3, which are cartilage-breaking enzymes, causing cartilage deterioration in RA (Mengshol et al. 2002; Ostrowska et al. 2018a). Also, the infiltration of synovial tissue induces the production of the nod-like receptor protein 3 (NLRP3) inflammasome, a key precursor of interleukin-1β (IL-1β) and interleukin-18 (IL-18), that augments synovial cell proliferation (Shin et al. 2019).

Interestingly, recent evidences have shown that inhibiting ferroptosis leads to decreased inflammation and defective synovial fibroblast proliferation, indicating a potential role for ferroptosis in the progression of RA (Feng et al. 2024; Liu et al. 2024). These previous investigations have indicated a potential treatment choice for reducing inflammation, joint damage and RA progression in patients by targeting vital ferroptotic pathways.

Ticagrelor is the first approved oral drug as a reversible P2Y12 inhibitor to prevent platelet aggregation in patients who are suffering from coronary artery diseases (Rollini et al. 2016). It elevates tissue adenosine levels, so it acts as an anti-inflammatory and inhibits oxidative stress (Barletta et al. 2012; Nylander et al. 2013). Previous researches indicated that ticagrelor's renoprotective effects were mediated by its ability to exert anti-inflammatory, antioxidant, and anti-apoptotic actions that maintain renal function and structure (El-Mokadem et al. 2021; Mansour et al. 2022). Additionally, ticagrelor showed a neuroprotective effect in Parkinsonism by inhibiting apoptosis (Muneeb et al. 2025). Moreover, ticagrelor mitigated cecal ligation and puncture (CLP)-induced polymicrobial sepsis in mice, likely through modulation of pro-inflammatory and oxidative stress signaling pathways (Mueen et al. 2023). Added to that, prior research proved that it could inhibit osteoclast differentiation and promote bone generation in a mice model (Mediero et al. 2016). Along with prior evidences, its potential effect on arthritis has not been previously discussed. Interestingly, in vitro, P2Y12 inhibition diminished inflammatory response and ferroptosis in iron-overloaded macrophages by outflowing excessive iron from macrophages into the blood circulation (Hu et al. 2024).

In light of this background, the current study aimed to investigate the possible novel anti-arthritic effect of ticagrelor in the adjuvant-induced arthritis (AIA) model in rats with the possible modulation of some inflammatory signals linked to ferroptotic cell death. In particular, the crosstalk between ferroptosis and NLRP3 was highlighted as a potential target for RA therapies.

## Materials and methods

### Animals

Male albino Wistar rats (200–250 g) were obtained from National Research in Cairo, Egypt. Regular feed and unlimited water were provided to the rats, which were housed in an air-conditioned environment with a temperature of 25 °C and alternating 12-h light and dark cycles. The animals were exposed to laboratory conditions for a week. The research designs and animal care were performed by the Guide for Laboratory Animals the Care and Use (NIH Publication, No. 8523, revised [2011]). Ethical approval for this study was obtained from Cairo University's Ethics Committee under license number (PT 3422, [2023]). (https://drive.google.com/file/d/1Q8GaUWYOK_nuNasZShPUloPNV3SChn4b/view?usp=sharing).

### Drugs and dosage

#### Induction

Freund’s Complete Adjuvant (FCA) purchased from (Sigma Aldrich., MO, USA) was injected in the sub-plantar of the left hind paw as a single dose of 0.1 ml (Zhu et al. 2020).

#### Drugs

Ticagrelor (AstraZeneca, Egypt) 30 mg/kg (Mansour et al. 2022) was given orally once a day throughout the study period (21 days) and it was dissociated in 4% dimethyl sulfoxide (DMSO) and subsequently diluted with normal saline (Oury et al. 2023).

### Experimental design

**Fig. 1:**
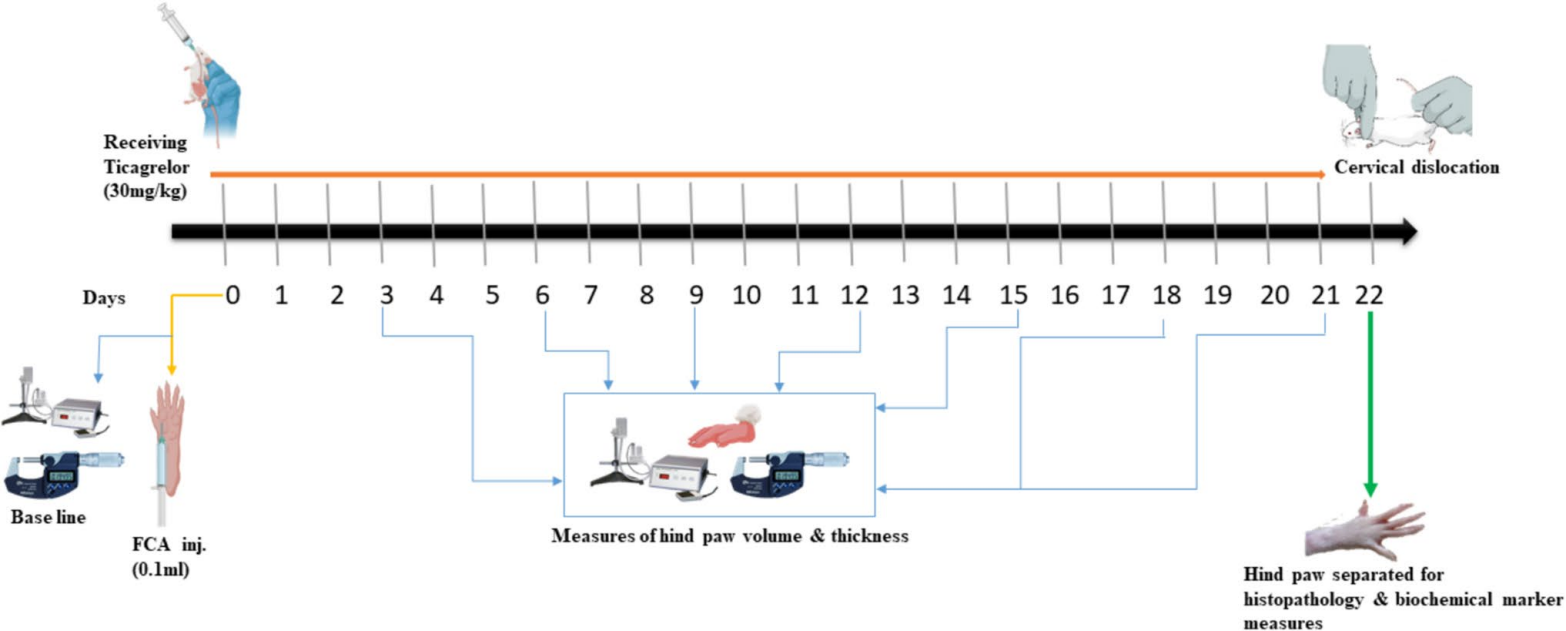
Experimental diagrammatic presentation: The yellow arrow represents FCA injection at day 0. The orange arrow represents the days of receiving ticagrelor (21 days). Blue arrows symbolize the days of measuring hind paw volume and thickness as well as arthritic score. The green arrow represents the end of the experiment, and adjuvant arthritis hind paws were separated for measuring biochemical markers, histopathology and immunohistochemistry

A total of 32 male albino Wistar rats were distributed randomly to 4 groups (n = 8 for each), the selection of n = 8 animals per group was indeed determined through a prospective power calculation by using G* power version 3.1.9.7 software with α (type-1 error) of 0.05, power of 0.8 and large effect size. They were handled as follows: Control group: rats received (4% DMSO diluted with normal saline) by oral gavage for 21 days; Ticagrelor group: rats received ticagrelor daily (30 mg/kg, p.o.) throughout the study period (21 days); AIA group: rats were injected in the sub-plantar of the left hind paw (0.1 ml FCA) as a single dose on day 0; and Ticagrelor + AIA group: rats received ticagrelor (30 mg/kg, p.o) following induction of AIA by injection in the sub-plantar of the left hind paw (0.1 ml FCA) (Fig. 1).

### Assessments of arthritis

Paw thickness and swelling volume of the left hind paw in each rat were assessed by digital micrometer (Mitutoyo, Japanese) and plethysmometer (Panlap, Harvard Apparatus, LE7500), respectively, at day 0 (baseline) and regular 3-day intervals for 21 days post-FCA injection. The extent of arthritis was determined visually using a standardized scoring system with a range of 0 to 4: (0, no swelling or redness; 1, slight swelling and/or redness; 2, mild swelling and redness; 3, significant swelling and redness with limited joint movement; 4, severe deformity and inability to use the limb) (El-Sheikh et al. 2019).

### Animal euthanization and tissue processing

At the end of the experiment on day 22, all of the animals were euthanized by decapitation under light anesthesia using thiopental sodium (5 mg/kg) (El-Abhar et al. 2018). Then the left hind paws of rats from each group were separated and parts of them were fixed in a 10% formalin solution for forty-eight hours, then paraffin embedding yielded paraffin blocks ready for histopathological examination and immunohistochemical analyses, while others were kept at -80°C to be used for biochemical analyses.

### Enzyme-linked immunosorbent analysis (ELISA)

Minces of ankle tissues were homogenized in ice- phosphate-buffered saline (ice-PBS) (usually 10 mg tissue in 100 µl of PBS), and then tissue homogenates were subjected according to the ELISA kit with the source and catalog number to estimate the following parameters: SLC7A11 (Biorbyt, Cat. No: orb781117, Cambridge, UK), GPX4 (MyBioSource, Cat. No: MBS069787, US), FTH1 (LSBio, Cat. No: LS-F33593, US), ACSL4 (MyBioSource, Cat. No: MBS3809786, US), ALOX15 (MyBioSource, Cat. No: MBS103451, US), and inflammatory chemokines like RANTES/CCL5 (R&D Systems Quantikine™ ELISA, MMR00, US), macrophage inflammatory protein-1α (MIP-1α)/CCL3 (Abcam, ab213916, Cambridge, UK), eotaxin-3/CCL26 (MyBioSource, Cat. No: MBS263571, US), and NLRP3 inflammasome (MyBioSource, Cat. No: MBS7255410, US). All the procedures were achieved under the manufacturer's instructions. The total protein concentration within the tissue homogenate was quantified following the methodology outlined by Bradford (Bradford 1976).

### Weston blot analysis

Total proteins were isolated from ankle tissues by Ready Prep™ protein extraction kit (Bio-Rad Inc., CA, USA), which were then evaluated via a bicinchoninic acid protein assay kit (Beyotime, Shanghai, China). Protein extracts in equal quantities were electro-transferred to a polyvinylidene fluoride membrane after being put onto an SDS-PAGE gel. Following blocking for one and a half hours at ambient temperature per 5% BSA in Tris-buffered saline and Tween 20, the isolated proteins underwent overnight incubation with primary antibodies at 4 °C. The primary antibodies included MMP13 (diluted (1:1,000), cat. no. 18165–1-AP, proteintech, California, USA.) and MMP3 (diluted (1:1,000), cat. no. ab52915; Abcam, Cambridge, UK). The protein-antibody complex was observed utilizing a secondary antibody HRP (Novus Biologicals, USA, Catalogue No. NB7160), which was diluted at 1:5000. The chemiluminescence was detected via an imager with a CCD camera. The β-actin band density was used to quantify protein bands using Image J, a free program from NIH in Bethesda, USA (RRID: SCR 003070).

### Histopathological examination

Specimens of bone, articular cartilage, and synovial membrane underwent fixation in 10% neutral buffered formalin; after that, they were decalcified in 10% EDTA, then trimmed, rinsed in water, dehydrated in rising grades of ethyl alcohol, rinsed in xylene, and placed in paraffin. Thin sections (4–6 µ) were processed and stained with Hematoxylin & Eosin stain (H&E) (Bancroft and Gamble 2008) as a general examination staining method, observed by a light microscope and examined by an independent pathologist. Mononuclear cell infiltration and pannus formation, which resulted in cartilage and bone destruction, were quantified using a defined scoring system. Infiltration was rated as (0, no infiltration; 1, mild infiltration; 2, moderate infiltration; 3, severe infiltration). Cartilage and bone destruction by pannus formation (0, no alteration; 1, mild alteration, pannus invasion within cartilage; 2, moderate alteration, pannus invasion into cartilage/subchondral bone; 3, severe alteration, pannus invasion into the subchondral bone) (Taniguchi et al. 1999).

### Immunohistochemistry

TNF-α and P53 in ankle tissue were analyzed by immunohistochemistry. The paraffin sections inserted into the paw tissue were immersed in xylene followed by rehydration using solutions of graded ethanol. The slides were then blocked for two hours, as needed, using either PBS (pH 7.4) or tris-buffered saline containing 1% bovine serum albumin. The avidinbiotin-peroxidase complex (ABC) method was employed for mounting them on positively charged slides, polyclonal anti-TNF-α (Abclonal, Cat. No. A11534, US, at dilution 1:1000) and polyclonal anti-P53 (Abclonal, Cat. No. A5761, US, at dilution 1:100). Following a 1-h incubation with these antibodies, sections from each group were processed using the ABC method (Vectastain ABC-HRP kit, Vector Laboratories) by addition of the essential reagent. They were rinsed out with PBS, then incubated with HRP-conjugated IgG secondary antibody (Abclonal, Cat. No. (AS014) at a 1:10,000 dilution). To detect the antigen–antibody complex, peroxidase was used to label marker expression and color with 0.02% diaminobenzidine (DAB, manufactured by Sigma), which included 0.01% H_2_O_2_. Negative controls were established by using non-immune serum rather than primary or secondary antibodies. Sections dyed with IHC were observed under an Olympus microscope (BX-63). For quantitative analysis, images were imported into ImageJ software (version 1.53t, Wayne Rasband and contributors, National Institutes of Health, USA). To quantify the DAB signal, images first underwent color deconvolution to separate the brown DAB chromogen. The resulting grayscale DAB channel was then subjected to thresholding to objectively define and isolate the positive staining for subsequent area percentage calculation.

### Statistical analysis

To evaluate the data’s adherence to normal distribution, the Shapiro–Wilk's test was performed, and the analysis was conducted using GraphPad Prism (version 9, GraphPad Software, Inc., San Diego, CA). All data were displayed as the mean ± SD. Two-way ANOVA followed by Tukey's test was employed to control the Family-Wise Error Rate (FWER) at α = 0.05 across all pairwise comparisons to ensures proper adjustment for multiple comparisons. Nevertheless, a Kruskal–Wallis test followed by a post-hoc Dunn’s test was performed for the non-parametric data. The effect sizes (η^2^ = SS effect /SS total) for the ANOVA were calculated to evaluate magnitude ticagrelors effect. (η^2^: large = 0.14, medium = 0.06, small = 0.01). At p ≤ 0.05, the significant difference was applied. Correlation analysis was accomplished using Pearson's correlation coefficient.

## Results

### Ticagrelor ameliorated macroscopic arthritis changes in AIA rat model

**Fig. 2:**
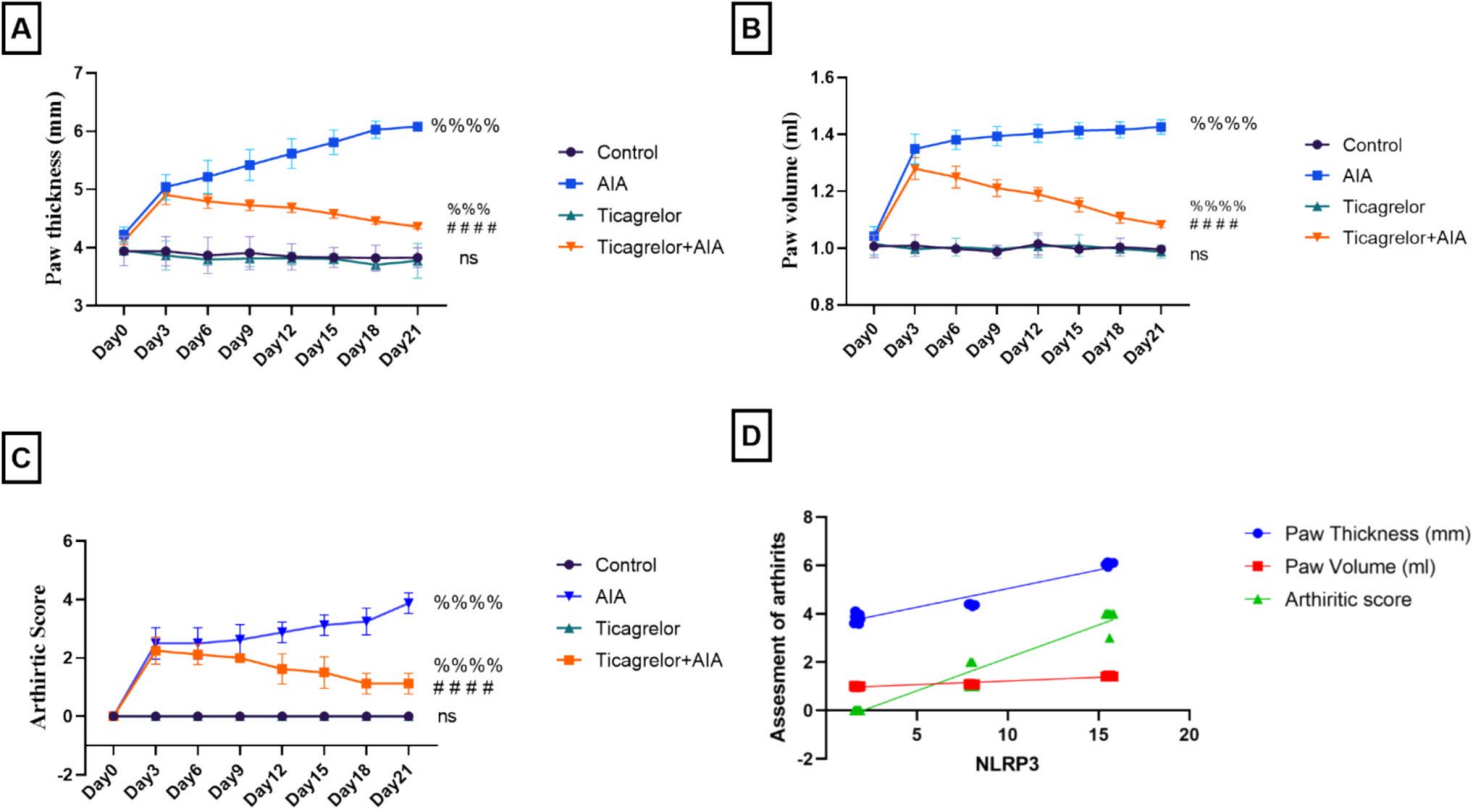
Effect of ticagrelor on macroscopic arthritis changes in the AIA rat model. **A** Paw thickness. **B** Paw volume. **C** Arthritic score. **D** Correlation analysis between NLRP3 and macroscopic arthritis changes. Results are provided as means ± SD; p ≤ 0.05. (ns) non-significance, ^%%%^ p < 0.001, ^%%%%^ p < 0.0001 significant difference compared to the control group, ^####^ p < 0.0001 significant difference compared to AIA group. Ticagrelor was given orally (30 mg/kg) for 21 days, AIA was induced by FCA (0.1 ml single dose S.C.). Two-way ANOVA with Tukey–Kramer post hoc testing was employed for statistical analysis. Correlation analysis was implemented based on Pearson’s correlation coefficient

Sub-plantar injection of FCA into the left hind paws of rats was associated with a significant elevation in paw thickness, paw volume and arthritic score in comparison with the control group. However, treatment with ticagrelor mitigated arthritis severity as revealed by the marked reduction in paw thickness, paw volume and arthritic score by 61.3%, 34.78%, and 71.79%, respectively, at day 21 compared to the untreated AIA group. Besides, the expression of NLRP3 was potently correlated positively with all macroscopic arthritis changes (r = 0.9635, [95% CI of difference 0.9162 to 0.9843, p < 0.0001], r = 0.9617, [95% CI of difference 0.9122 to 0.9835, p < 0.0001] and r = 0.9756, [ 95% CI of difference 0.9436 to 0.9896, p < 0.0001], respectively) (Fig. 2A–D).

### Ticagrelor boosted ferroptosis-inhibiting factors in the AIA rat model

**Fig. 3:**
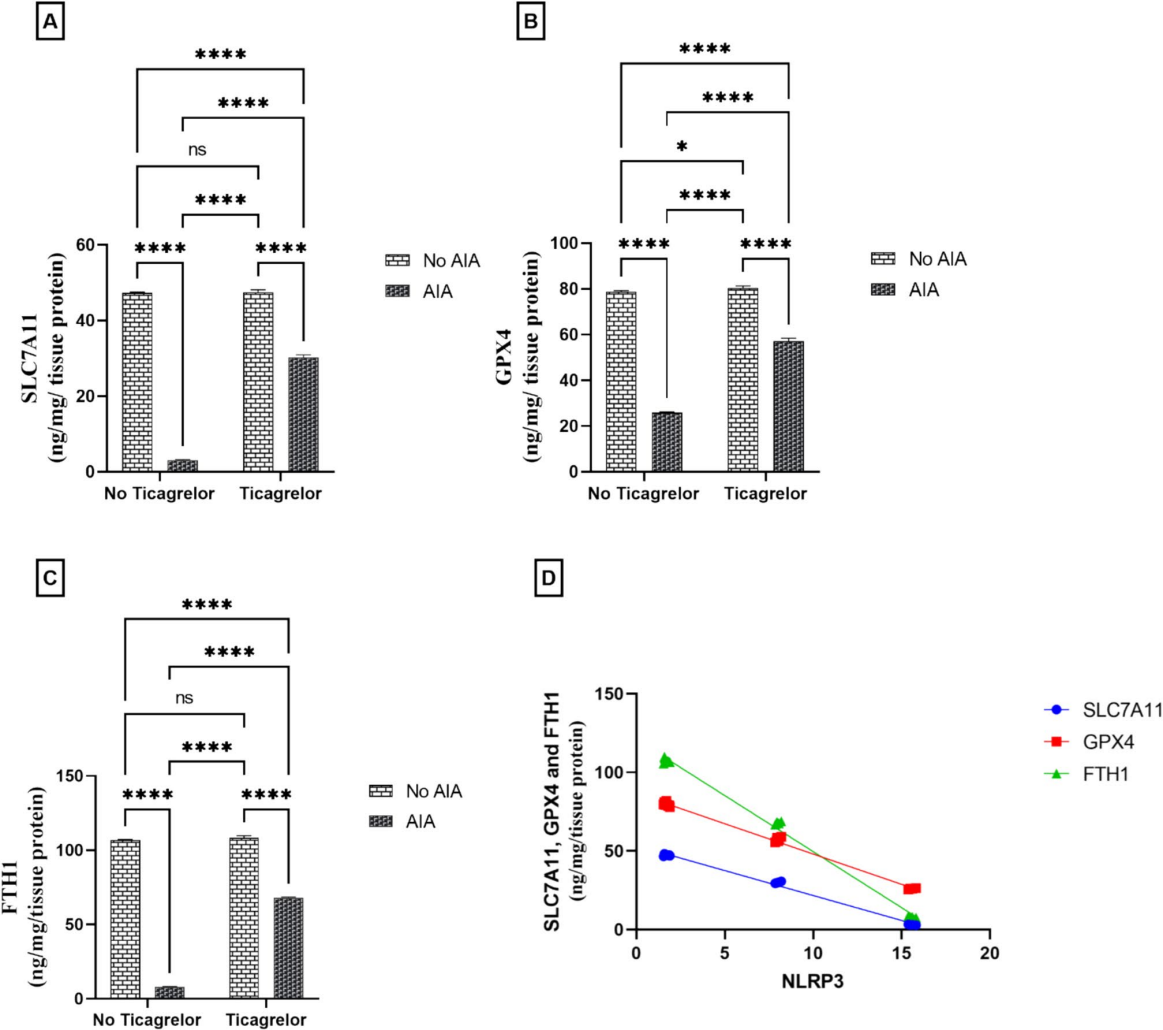
Effect of ticagrelor on ferroptosis inhibiting factors in the AIA rat model. **A** SLC7A11. **B** GPX4. **C** FTH1 using ELISA analysis. First bar represents the control group, Second Bar represents the AIA group, Third Bar represents the Ticagrelor group and Fourth Bar represents the Ticagrelor + AIA group. **D** Correlation analysis between NLRP3 and ferroptosis inhibiting factors. Ticagrelor was given orally (30 mg/kg) for 21 days, AIA was induced by FCA (0.1 ml single dose S.C.). The left hind paws of rats from each group were separated on (day 22) then kept at - 80°C for SLC7A11, GPX4 and FTH1 ELISA analysis. Two-way ANOVA with Tukey–Kramer post hoc testing was employed for statistical analysis. Data are provided as means ± SD; p ≤ 0.05. (ns) non-significance, * p < 0.05, **** p < 0.0001. Correlation analysis was implemented based on Pearson’s correlation coefficient

A marked decrease in SLC7A11 (F_(1, 20)_ = 25,932, p < 0.0001), GPX4 (F_(1, 20)_ = 11,568, p < 0.0001), and FTH1 (F_(1, 20)_ = 35,123, p < 0.0001) contents by 93.47%, 67.11%, and 92.51%, respectively, was detected in the AIA group rats compared to the control group. The mean difference between the control and AIA group for SLC7A11 was (44.24, [ 95% CI of difference 43.49 to 45.00, adjusted p < 0.0001]), GPX4 (52.88, [95% CI of difference 51.48 to 54.28, adjusted p < 0.0001]) and FTH1(98.85, [95% CI of difference 97.38 to 100.3, adjusted p < 0.0001]). Receiving ticagrelor modulated ferroptosis in FCA-induced arthritis rats via upregulation of SLC7A11 (F_(1, 20)_ = F_(1, 20)_ = 5152, p < 0.0001, η^2^ = 0.16) GPX4 (F_(1, 20)_ = 2150, p < 0.0001, η^2^ = 0.15) and FTH1 (F_(1, 20)_ = 6806, p < 0.0001, η^2^ = 0.16) content to reach 9.80, 2.20, and 8.49-folds compared to the AIA group rats. Interactions were recorded between the two factors (AIA and ticagrelor) for SLC7A11 (F_(1, 20)_ = 5009, p < 0.0001), GPX4 (F_(1, 20)_ = 1737, p < 0.0001) and FTH1 (F_(1, 20)_ = 6185, p < 0.0001). The mean difference between the AIA group and Ticagrelor + AIA group for SLC7A11 was (− 27.21, [95% CI of difference − 27.96 to − 26.45, adjusted p < 0.0001]), GPX4 (− 31.20, [95% CI of difference − 32.60 to − 29.80, adjusted p < 0.0001]) and FTH1(− 59.87, [95% CI of difference − 61.34 to − 58.40, adjusted p < 0.0001]) Notably, NLRP3 expression was significantly and negatively correlated with ferroptosis inhibiting factors (r = − 0.9976, [95% CI − 0.9990 to − 0.9943, p < 0.0001], r = − 0.9982, [95% CI − 0.9993 to − 0.9959, p < 0.0001] and r = − 0.9980, [95% CI − 0.9991 to − 0.9952, p < 0.0001], in the same way) (Fig. 3A–D).

### Ticagrelor repressed ferroptosis-promoting factors in the AIA rat model

**Fig. 4:**
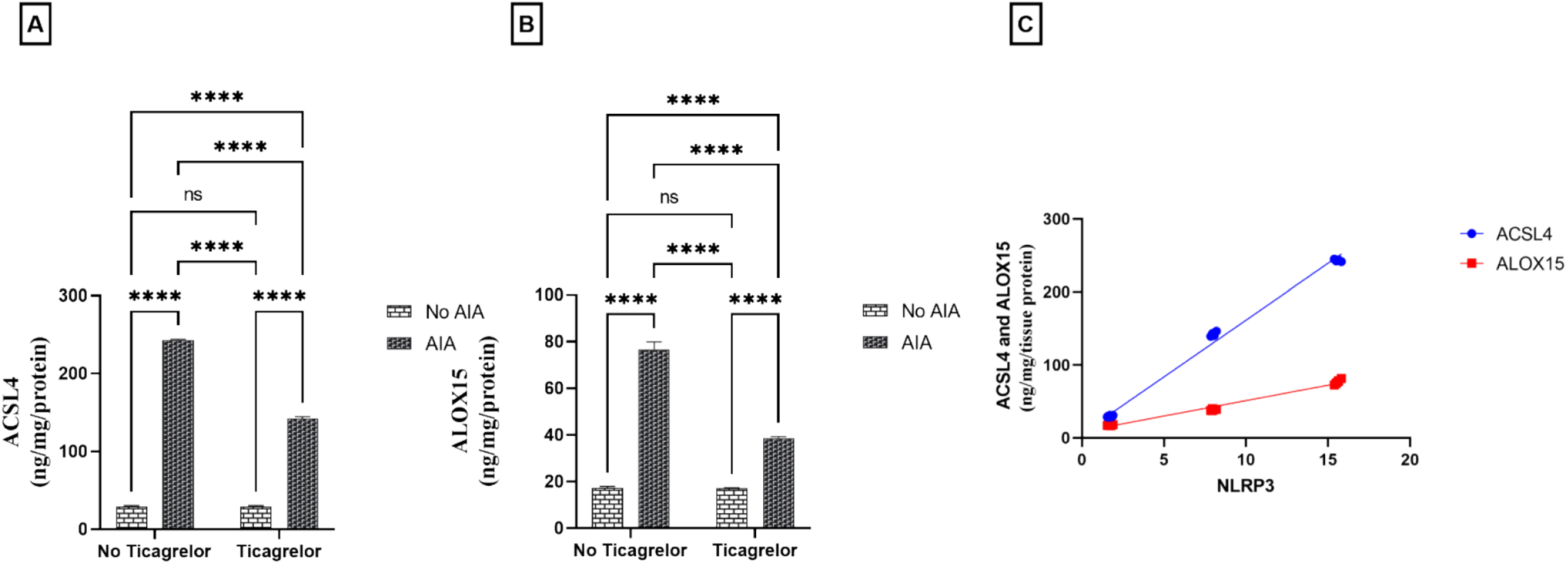
Effect of ticagrelor on ferroptosis-promoting factors in the AIA rat model. **A** ACSL4 **B** ALOX15 using ELISA analysis. First Bar represents the control group, Second Bar represents the AIA group, Third Bar represents the Ticagrelor group and Fourth Bar represents the Ticagrelor + AIA group. **C** Correlation analysis between NLRP3 and ferroptosis-promoting factors. Ticagrelor was given orally (30 mg/kg) for 21 days, AIA was induced by FCA (0.1 ml single dose S.C.). The left hind paws of rats from each group were separated on (day 22) and then kept at – 80 °C for ACSL4 and ALOX15 ELISA analysis. Two-way ANOVA with Tukey–Kramer post hoc testing was employed for statistical analysis. Results are provided as means ± SD; p ≤ 0.05. (ns) non-significance, **** p < 0. 0001. Correlation analysis was implemented based on Pearson’s correlation coefficient

ACSL4 and ALOX15 are critical mediators of lipid peroxidation during ferroptosis. ACSL4 (F_(1, 20)_ = 65,925, p < 0.0001), and ALOX15 (F_(1, 20)_ = 3441, p < 0.0001) levels were significantly raised in AIA rats to 8.29 and 4.42-folds, respectively, compared to the control group. The mean difference between the control and the AIA group for ACSL4 was (− 213.7, [95% CI of difference − 216.2 to − 211.2, adjusted p < 0.0001]) and ALOX15 (− 59.34, [95% CI of difference − 62.06 to − 56.62, adjusted p < 0.0001]). Receiving ticagrelor throughout the experiment led to a marked decrease in both ACSL4 (F_(1, 20)_ = 6267, p < 0.0001, η^2^ = 0.08) and ALOX15 (F_(1, 20)_ = 780.0, p < 0.0001, η^2^ = 0.18) levels by 41.45% and 49.85%, respectively, compared to the AIA group. The mean difference between the AIA group and Ticagrelor + AIA group for ACSL4 was (100.8 [95% CI of difference 98.23 to 103.3, adjusted p < 0.0001]), ALOX15 (38.22, [95% CI of difference 35.50 to 40.94, adjusted p < 0.0001]). Interactions were recorded between the two factors (AIA and ticagrelor) for ACSL4 (F_(1, 20)_ = 6288, p < 0.0001) and ALOX15 (F_(1, 20)_ = 766.9, p < 0.0001) Also, a potent positive correlation was described between NLPR3 expression and ferroptotic promoters (r = 0.9968 [95% CI of difference 0.9924 to 0.9986, p < 0.0001] and r = 0.9934 [95% CI of difference 0.9846 to 0.9972, p < 0.0001], correspondingly) (Fig. 4A–C).

**Fig. 5:**
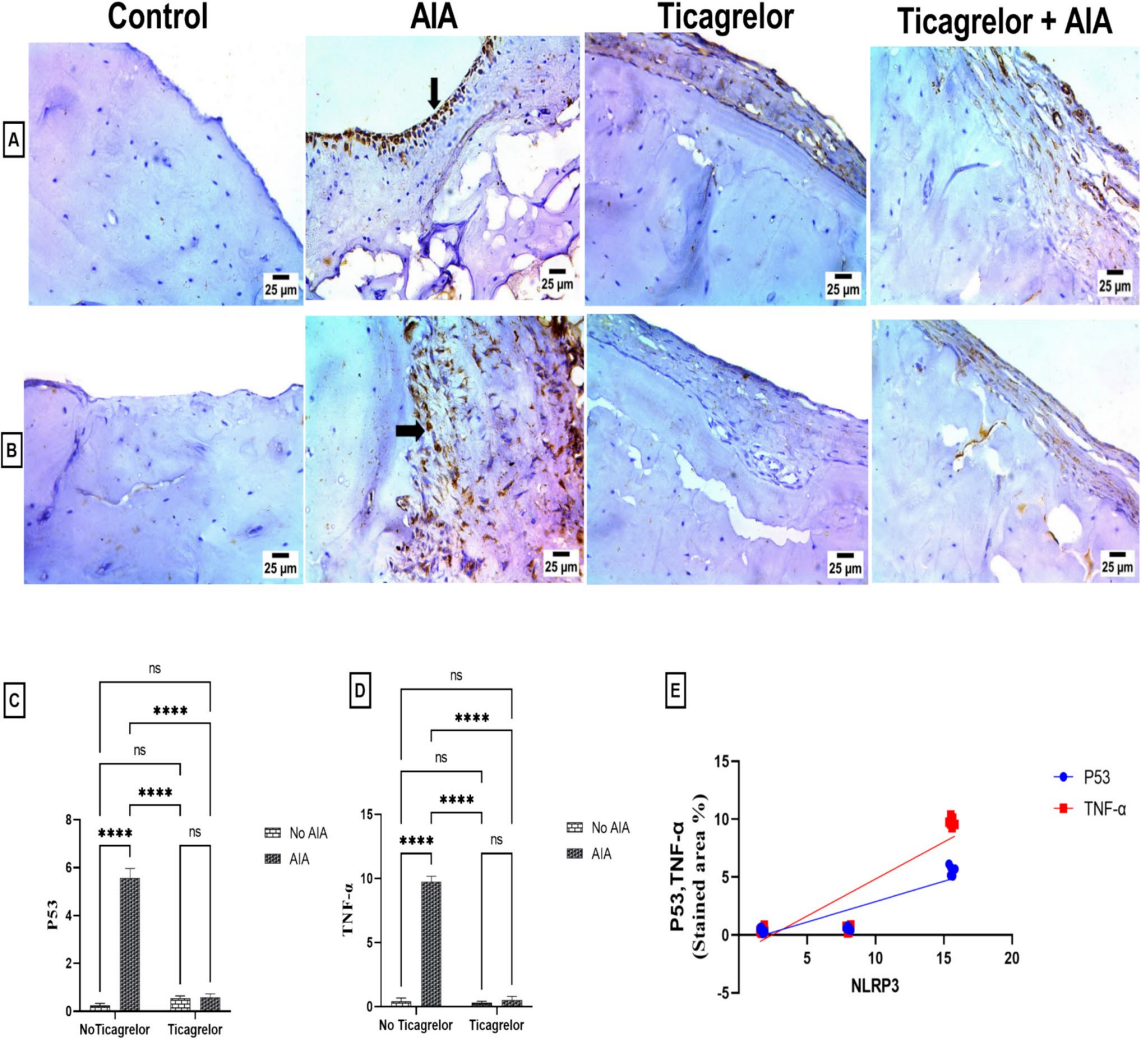
Effect of ticagrelor on P53 and TNF-α in AIA rat model. **A** photomicrograph of IHC-Peroxidase-DAB immunostaining of P53 in bone (40 ×), (arrow) shows sever positive expression for P53 in bone cells. **B** photomicrograph of IHC-Peroxidase-DAB immunostaining of TNF-α in bone (40 ×), (arrow) shows sever positive expression for TNF-α in fibrous tissue of bone. **C** Bar graph represents P53 staining area %. **D** Bar graph of TNF-α staining area %. First Bar represents the control group, Second Bar represents the AIA group, Third Bar represents Ticagrelor group and Fourth Bar represents the Ticagrelor + AIA group. **E** Correlation analysis between NLRP3 and P53 as well as TNF-α. Ticagrelor was given orally (30 mg/kg) for 21 days, AIA was induced by FCA (0.1 ml single dose S.C.). Specimens of bone underwent fixation in 10% neutral buffered formalin; after that, they were decalcified in 10% EDTA for immunohistochemistry analysis. Two-way ANOVA with Tukey–Kramer post hoc testing was employed for statistical analysis. Results are provided as means ± SD; p ≤ 0.05. (ns) non-significance, ****p < 0.0001. Correlation analysis was implemented based on Pearson’s correlation coefficient

Furthermore, immunohistochemical examination revealed a prominent upsurge in the bone expression of P53 (F_(1, 20)_ = 887.3, p < 0.0001) following FCA administration, reaching 23.03-fold the control group. The mean difference between the control and the AIA group for P53 was (− 5.325 [95% CI of difference − 5.682 to − 4.968, adjusted p < 0.0001]. On the other hand, ticagrelor was effective in suppressing its expression (F_(1, 20)_ = 674, p < 0.0001, η^2^ = 0.4) by 89.46% as compared to AIA rats. The mean difference between the AIA group and Ticagrelor + AIA group for P53 was (4.980 [95% CI of difference 4.623 to 5.337, adjusted p < 0.0001]). Interactions were noted between two factors (AIA and ticagrelor) for P53 (F_(1, 20)_ = 856.8, p < 0.0001). Furthermore, the expression of NLRP3 was strong positively correlated with the P53-stained area % (r = 0.9027 [95% CI of difference 0.7852 to 0.9574, p < 0.0001]) (Fig. 5A, C and E).

### Ticagrelor hampered the expression of TNF-α and pro-inflammatory chemokines in the AIA rat model

In the AIA group, the immunohistochemical analysis showed a significant upregulation of TNF-α expression in bone (F_(1, 20)_ = 1723, p < 0.0001) to 22.87-fold compared with the control group. The mean difference between the control and the AIA group for TNF-α was (− 9.335 [95% CI of difference − 9.791 to − 8.879, adjusted p < 0.0001]. Contrariwise, it was markedly downregulated (F_(1, 20)_ = 1649, p < 0.0001, η^2^ = 0.48) with receiving ticagrelor that hampered the FCA effect by 94.6% compared to the AIA group. The mean difference between the AIA group and Ticagrelor + AIA group for TNF-α was (9.232 [95% CI of difference 8.776 to 9.688, adjusted p < 0.0001]). Interactions were recorded between two factors (AIA and ticagrelor) for TNF-α (F_(1, 20)_ = 1563, p < 0.0001). Likewise, there was a strong positive correlation between NLRP3 expression and TNF-α stained area% (r = 0.8984, [95% CI of difference 0.7763 to 0.9555, p < 0.0001]) (Fig. 5B, D and E).

**Fig. 6:**
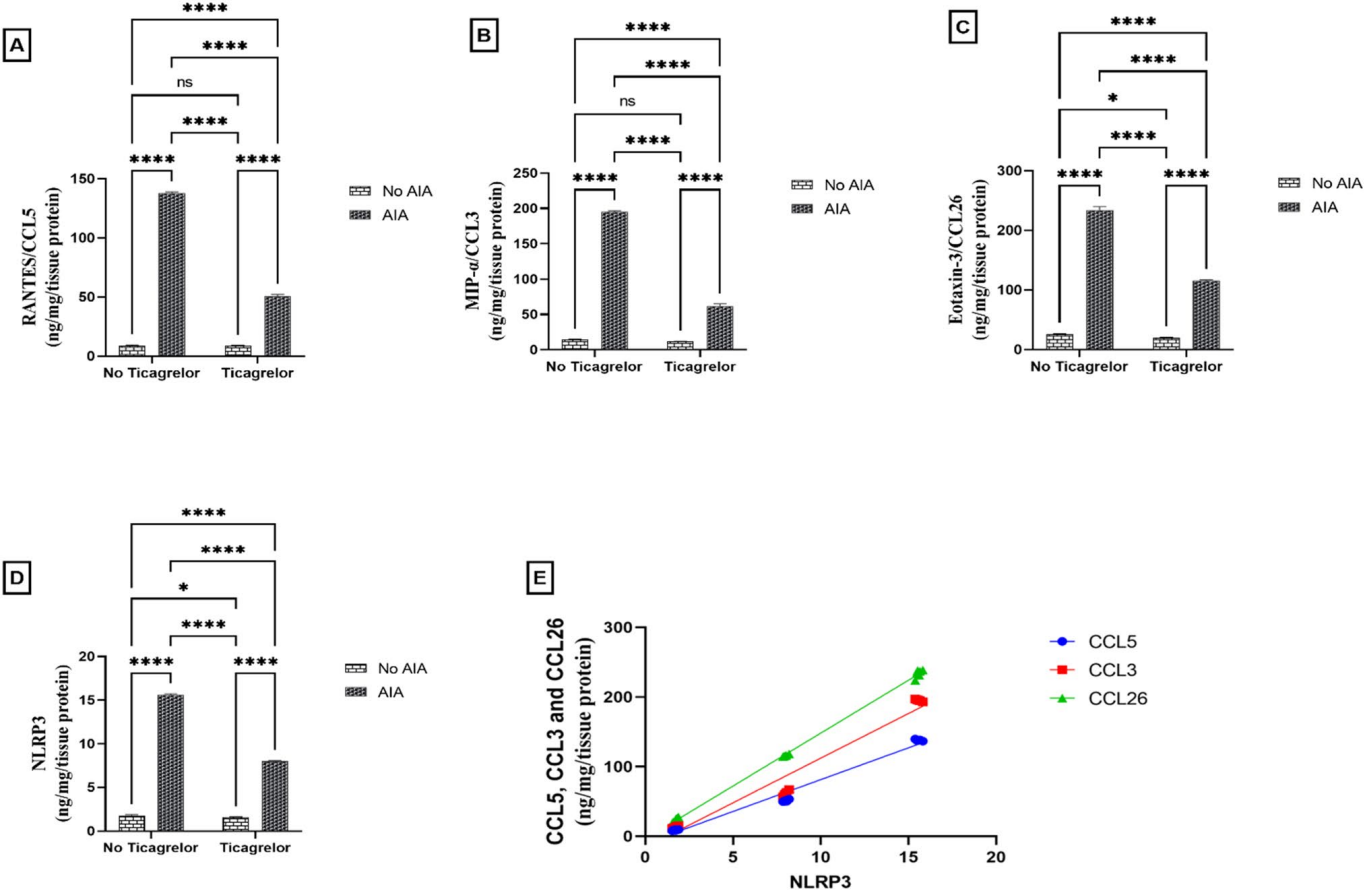
Effect of ticagrelor on pro-inflammatory chemokines and NLRP3 in AIA rat model. **A** RANTES/CCL5. **B** MIP-1α/CCL3, **C** Eotaxin-3 /CCL26 **D** NLRP3 using ELISA analysis. First Bar represents the control group, Second Bar represents the AIA group, Third Bar represents the Ticagrelor group and Fourth Bar represents the Ticagrelor + AIA group. **E** Correlation analysis between NLRP3 and all chemokine markers. Ticagrelor was given orally (30 mg/kg) for 21 days, AIA was induced by FCA (0.1 ml single dose S.C.). The left hind paws of rats from each group were separated on (day 22) then kept at -80°C for RANTES/CCL5, MIP-1α/CCL3, Eotaxin-3/CCL26 and NLRP3 ELISA analysis. Two-way ANOVA with Tukey-Kramer post hoc testing was employed for statistical analysis. Data are provided as means ± SD; p ≤ 0.05. (ns) non-significance, *p < 0.05. ****p < 0.0001. Correlation analysis was implemented based on Pearson’s correlation coefficient

RANTES/CCL5, MIP-1α/CCL3, and eotaxin-3/CCL26 are chemokines that attract white blood cells and amplify inflammatory mediators. The content of RANTES/CCL5 (F_(1, 20)_ = 52,803, p < 0.0001), MIP-1α/CCL3 (F_(1, 20)_ = 19,616, p < 0.0001), and eotaxin-3/CCL26 (F_(1, 20)_ = 15,945, p < 0.0001) were significantly elevated to 15.13, 13.9, and 9.03-folds, respectively, in the AIA group compared with the control group. The mean difference between the control and the AIA group for RANTES/CCL5 was (− 128.8 [95% CI of difference − 130.3 to − 127.4, adjusted p < 0.0001], for MIP-1α/CCL3 was (− 181.2, [95% CI of difference − 184.5 to − 177.9, adjusted p < 0.0001] and for eotaxin-3/CCL26 was (− 208.0 [95% CI of difference − 212.8 to − 203.3, adjusted p < 0.0001]. In contrast, pretreatment with ticagrelor led to a significant decline in RANTES/ CCL5 (F_(1, 20)_ = 13,760, p < 0.0001, η^2^ = 0.2), MIP-1α/CCL3 (F_(1, 20)_ = 6835, p < 0.0001, η^2^ = 0.25), and eotaxin-3/CCL26 (F_(1, 20)_ = 2669, p < 0.0001, η^2^ = 0.14) content by 63.01%, 68.58%, and 50.58%, respectively, compared to AIA group. The mean difference between the AIA group and Ticagrelor + AIA group for RANTES/CCL5 was (86.93 [95% CI of difference 85.45 to 88.40, adjusted p < 0.0001]), for MIP-1α/CCL3 was (133.9, [95% CI of difference 130.6 to 137.2, adjusted p < 0.0001] and for eotaxin-3/CCL26 was (118.3 [95% CI of difference 113.5 to 123.1, adjusted p < 0.0001]. Interactions were recorded between two factors (AIA and ticagrelor) for RANTES/CCL5 (F_(1, 20)_ = 13,472, p < 0.0001), MIP-1α/CCL3 (F_(1, 20)_ = 6363, p < 0.0001), and eotaxin-3/CCL26 (F_(1, 20)_ = 2177, p < 0.0001). Also, a powerful positive correlation was observed between NLRP3 expression and all chemokines (r = 0.9907, [95% CI of difference 0.9783 to 0.9960, p < 0.0001], r = 0.9801, [95% CI of difference 0.9538 to 0.9915, p < 0.0001] and r = 0.9995, [95% CI of difference 0.9989 to 0.9998, p < 0.0001], respectively) (Fig. 6A–C, E).

### Ticagrelor diminished NLRP3 expression in the AIA rat model

As illustrated in (Fig. 6D), in the rats injected with FCA, the NLRP3 expression exhibited statistically significant upregulation (F_(1, 20)_ = 57,877, p < 0.0001) to 8.58-fold in comparison to the control. The mean difference between the control and AIA group for NLRP3 was (− 13.76 [95% CI of difference − 13.93 to − 13.60, adjusted p < 0.0001]. Conversely, rats that were pretreated with ticagrelor exhibited a marked decline in NLRP3 (F_(1, 20)_ = 8622, p < 0.0001, η^2^ = 0.13) by 48.63% relative to the AIA rats. The mean difference between the AIA group and Ticagrelor + AIA group for NLRP3 was (7.574 [95% CI of difference 7.408 to 7.740, adjusted p < 0.0001]). The interaction was recorded between two factors (AIA and ticagrelor) for NLRP3 (F_(1, 20)_ = 7735, p < 0.0001).

### Ticagrelor mitigated MMP13 and MMP3 expression AIA rat model

**Fig. 7:**
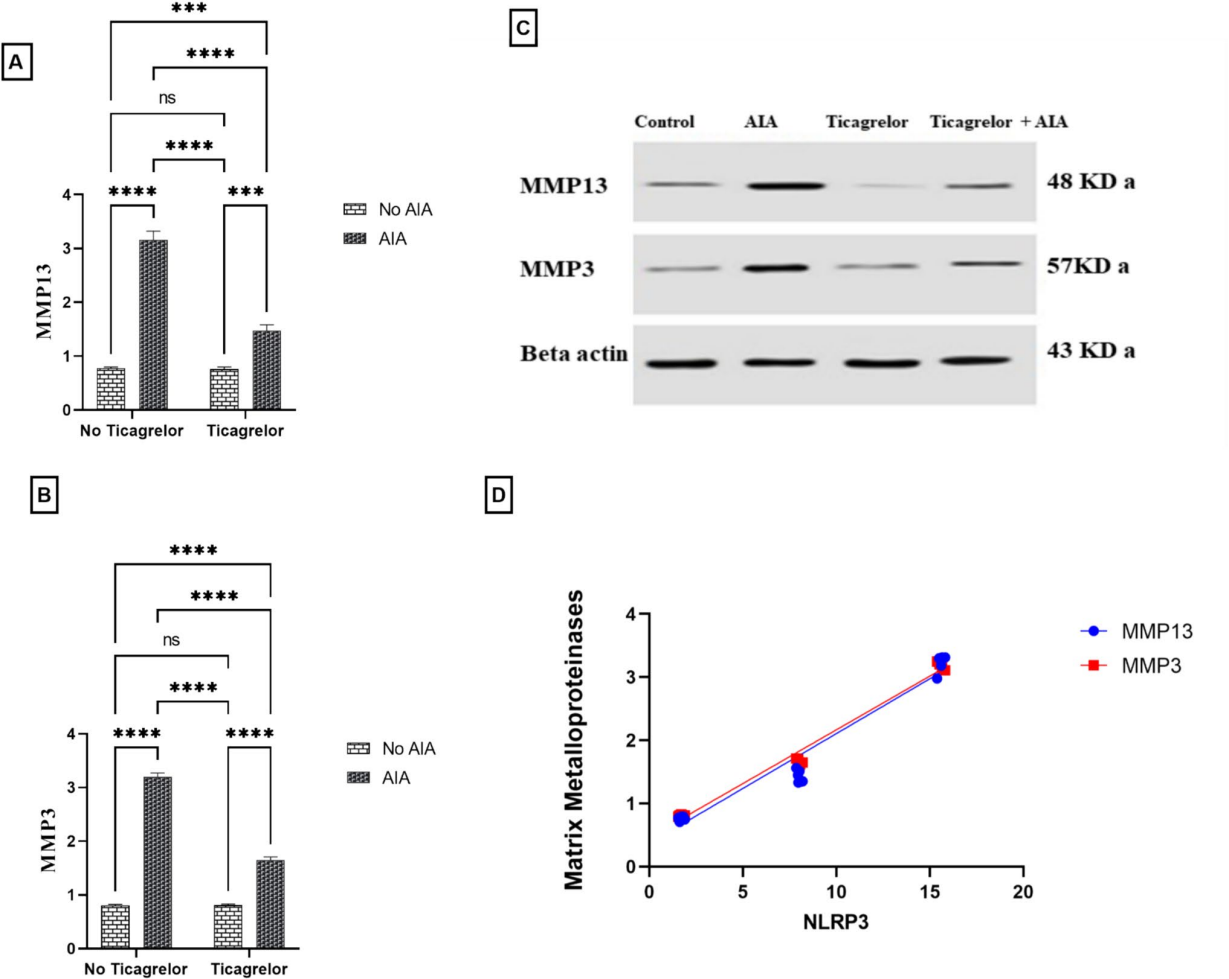
Effect of ticagrelor on MMP13 and MMP3 expression in the AIA rat model. **A** MMP13. **B** MMP3, First Bar represents the control group, Second Bar represents the AIA group, Third Bar represents the Ticagrelor group and Fourth Bar represents the Ticagrelor + AIA group. **C** Bands of western blot for MMP13 and MMP3 expressions within the left hind paw. **D** Correlation analysis between NLRP3 and matrix metalloproteinases. To verify the equivalent protein loading, β-actin levels were assessed. Ticagrelor was given orally (30 mg/kg) for 21 days, AIA was induced by FCA (0.1 ml single dose S.C.). The left hind paws of rats from each group were separated on (day 22) and then kept at – 80 °C for MMP13 and MMP3 western blot analysis. Two-way ANOVA with Tukey–Kramer post hoc testing was employed for statistical analysis. Results are provided as means ± SD; p ≤ 0.05. (ns) non-significance, **** p < 0.0001. Correlation analysis was implemented based on Pearson’s correlation coefficient

Measuring MMP13 and MMP3 is another mechanism that showed the potency of ticagrelor in modulating inflammation. MMP13 (F_(1, 8)_ = 686.0, p < 0.0001) and MMP3 (F_(1, 8)_ = 3274, p < 0.0001) were markedly upregulated in the AIA group compared to the control to reach 4.20 and 3.97-folds, respectively. The mean difference between the control and AIA group for MMP13 was (− 2.380 [95% CI of difference − 2.648 to − 2.112, adjusted p < 0.0001] and for MMP3 was (− 2.393 [95% CI of difference − 2.521 to − 2.265, adjusted p < 0.0001]. On the other hand, receiving ticagrelor hindered FCA effect by decreasing MMP13 (F_(1, 8)_ = 207.6, p < 0.0001, η^2^ = 0.23) and MMP3 (F_(1, 8)_ = 737.9, p < 0.0001, η^2^ = 0.18) expressions compared to the AIA group by 55.91% and 48.33%, respectively. The mean difference between the AIA group and Ticagrelor + AIA group for MMP13 was (1.683 [95% CI of difference 1.416 to 1.951, adjusted p < 0.0001] and for MMP3 was (1.543 [95% CI of difference 1.415 to 1.671, adjusted p < 0.0001]. Interactions were recorded between (AIA and ticagrelor) for MMP13 (F_(1, 20)_ = 197.9, p < 0.0001) and MMP3 (F_(1, 8)_ = 750.8, p < 0.0001). Added to that, there was a positive correlation between NLRP3 expression and both MMP13 and MMP3 (r = 0.9782 [95% CI was 0.9495 to 0.9907, p < 0.0001] and r = 0.9933, [95% CI was 0.9843 to 0.9971, p < 0.0001], respectively) (Fig. 7A–D).

### Effect of ticagrelor on the histological structure of bone, articular cartilage, and synovial membrane of paw tissue

**Fig. 8:**
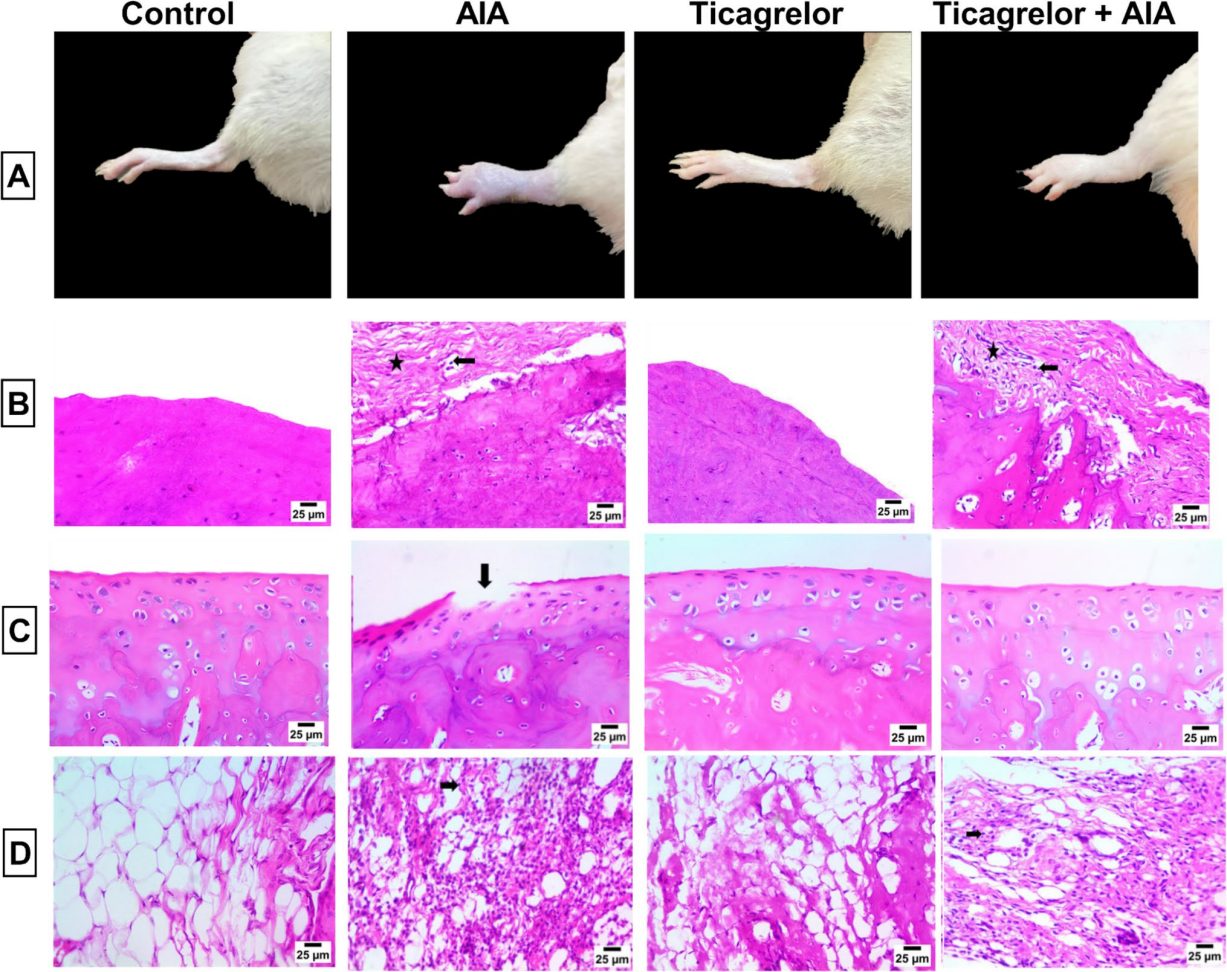
Effect of ticagrelor on the histological structure of bone, articular cartilage, and synovial membrane of paw tissue in AIA rat model. **A** Descriptive photographs of the ankle joint of rats. **B** H&E stain of bone, (star) displaying severe bone fibrosis and (arrow) indicating infiltration by a severe amount of mononuclear inflammatory cells in AIA group, on the other hand, (star) shows moderate bone fibrosis and (arrow) shows infiltration by few numbers of mononuclear inflammatory cells in the Ticagrelor + AIA group. **C** H&E stain of the articular cartilage surface, (arrow) shows a destruction and roughness of articular surface. **D** H&E stain of the synovial membrane, (arrow) displays infiltration of the synovial membrane by the high number of mononuclear inflammatory cells in AIA group, However, (arrow) expresses infiltration of synovial membrane by few numbers of mononuclear inflammatory cells in the Ticagrelor + AIA group. Ticagrelor was given orally (30 mg/kg) for 21 days, AIA was induced by FCA (0.1 ml single dose S.C.). Specimens of bone, articular cartilage, and synovial membrane underwent fixation in 10% neutral buffered formalin; after that, they were decalcified in 10% EDTA for histopathology analysis

Under microscopic examination, paw rats in the control group appeared to have a normal structure of bone, articular cartilage, and synovial membrane. The AIA group showed extensive bone fibrosis, accompanied by a severe amount of mononuclear inflammatory cells (Fig. 8B) associated with destruction and roughness of the articular cartilage surface (Fig. 8C), and high infiltration of inflammatory cells in the synovial membrane (Fig. 8D).

Interestingly, ticagrelor administration showed an anti-arthritic effect due to its ability to reduce the bone fibrosis (Fig. 8B), and the destruction of cartilage (Fig. 8C), along with hindering the infiltration of the synovial membrane in Ticagrelor + AIA group (Fig. 8D).

**Fig. 9:**
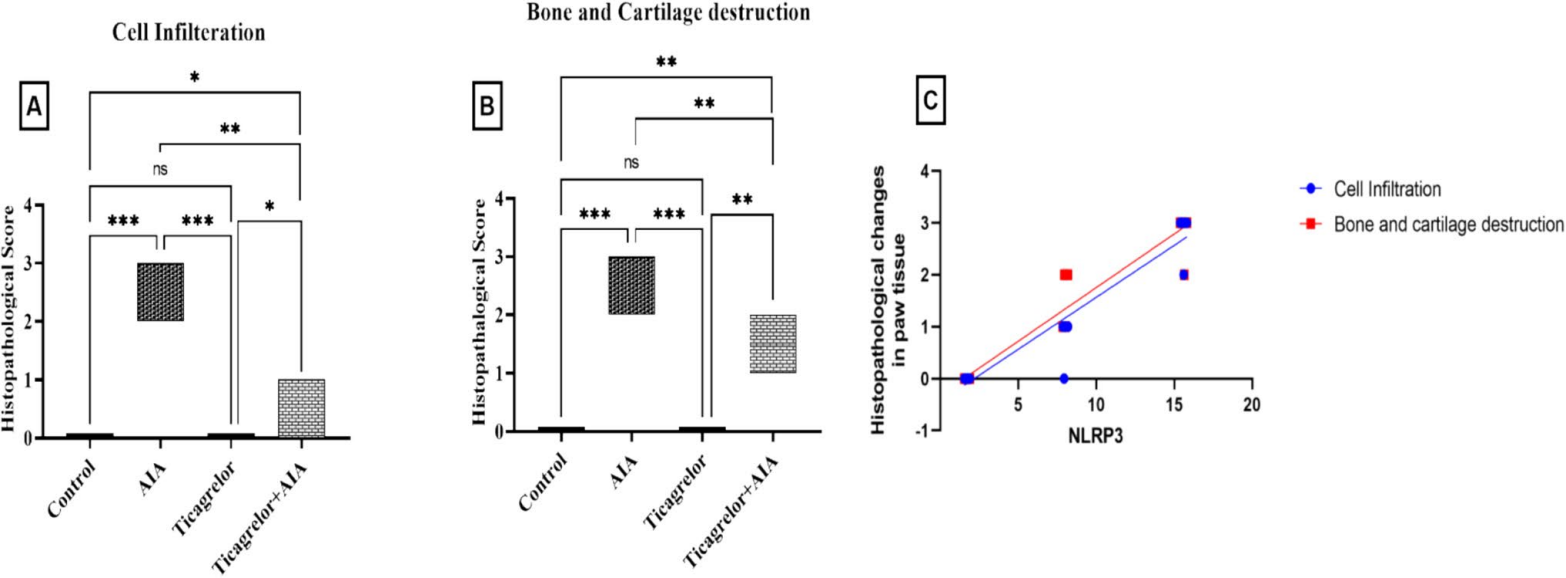
Effect of ticagrelor on histopathological alteration in paw tissue in AIA rat model. **A** Histopathological changes score of cell infiltration **B** Histopathological changes score of bone and articular destruction **C** Correlation analysis between NLRP3 and histopathological alteration in paw tissue. Data are represented as a box plot of the median. Kruskal–Wallis test was performed, followed by a post-hoc Dunn’s test for statistical analysis, (ns) non-significance. *p < 0.05, **p < 0.01, ***p < 0.001. Correlation analysis was implemented based on Pearson’s correlation coefficient

The scoring of histopathological alterations of paw rats for all groups is depicted in (Fig. 9A and 9B). In addition, we noted a strong positive correlation between NLRP3 expression and histopathological changes in paw tissue namely: cells infilteration (r = 0.9613, [95% CI was 0.9113 to 0.9834, p < 0.0001], and bone and cartilage destruction (r = 0.9645, [ 95% CI was 0.9185 to 0.9848, p < 0.0001], respectively) (Fig. 9C).

## Discussion

In our study, ticagrelor modulated P53/SL7A11/ALOX15 axis to attenuate inflammatory response linked to ferroptosis in the AIA rat model.

To date, there is no complete treatment for RA that has been established in humans and the typically administered drugs just help in mitigating its serious symptoms (Aletaha and Smolen 2018). Therefore, there is a critical need for the discovery of future therapy approaches. For the first time, our research focuses on evaluating the anti-inflammatory and anti-ferroptotic effects of ticagrelor against AIA.

The AIA is an experimental paradigm that simulates the pathophysiology of human RA, including chronic joint swelling and pain, pro-inflammatory cytokine release, cartilage erosion, and bone destruction. While it mimics many downstream inflammatory and destructive processes, it does not typically involve the persistent, self-perpetuating loss of self-tolerance or the robust production of specific autoantibodies seen in human RA (Jones et al. 2018). However, it is wieldy used to bio-screen new drugs for RA (El-Sayed et al. 2014; Zhu et al. 2020). We had been used male rats based on three key considerations: First, the historical precedent in scientific research, where male animals have traditionally been used to minimize variability introduced by hormonal fluctuations. Second, and more critically, estrogen has well-documented anti-inflammatory effects (Camporez et al. 2019) which could confound our investigation into the specific role of ticagrelor in AIA-related inflammation. Third, estrogen is an important hormone in the regulation of bone turnover and bone cell activity (Lara-Castillo 2021). By using male rats exclusively, we aimed to reduce this potential hormonal interference and ensure a more controlled assessment of the inflammatory response and the damage in the experimental arthritis model. Additionally, (Schuh et al. 2023) in their investigation of collagen induced arthritis (CIA) in middle-aged mice, observed an earlier onset of arthritis and more severe consequences on joints, bones, and kidneys in male mice compared to females. This finding highlights that sex is a crucial biological variable influencing disease severity and progression in inflammatory arthritis models. While the specific immune mechanisms underlying these sex differences and the limited duration of symptoms require further investigation, the demonstration of more severe disease outcomes in males supports our strategy of utilizing male rats to ensure a consistently robust and uniform disease phenotype for evaluating therapeutic interventions.

Ticagrelor administration showed a significant diminution in paw volume, thickness, and arthritic score in AIA rats that was confirmed in the histopathological examination. It ameliorated bone fibrosis and destruction of cartilage by reducing the number of infiltrated mononuclear inflammatory cells within the bone and synovial membrane. These findings demonstrate ticagrelor's efficacy in attenuating disease activity in the AIA model, suggesting its potential to alleviate pain and improve mobility in affected individuals.

Ferroptosis in RA has been linked to disrupted iron metabolism. The synovial fluid and membrane of RA patients with severe disease activity showed elevated levels of iron and lipid oxidation (Wu et al. 2022). A previous study has indicated decreased FTH expression by erastin that induced ferroptosis in RA (Zhou et al. 2022), which, besides its role in iron storage, has ferroxidase activity inhibiting ROS generation by converting ferrous to ferric iron (Mesquita et al. 2020). This observation is in harmony with our results where AIA rats showed a marked decline in FTH1 expression signifying the contribution of iron metabolism in inducing ferroptosis in RA.

One promising strategy for reducing ferroptosis is to enhance SLC7A11 levels which is indispensable for GSH synthesis, an essential co-activator for GPX4, the most significant anti-lipid peroxidase (Miao et al. 2022). Inactivation of GPX4 induced by GSH depletion secondary to SLC7A11 suppression eventually causes ferroptosis (Imai et al. 2017; Seibt et al. 2019). In this study, decreased expression of SLC7A11 and GPX4 was detected in AIA rats. Coherent with these results, previous studies showed a decrease in SLC7A11 and GPX4 levels enhancing ferroptosis in human synovial cells and AIA rats model (Luo and Zhang 2021; Peng et al. 2022). In a similar fashion, a marked diminution in GPX4 content was detected in the articular cartilage of RA patients (Zhou et al. 2022).

Emerging evidences reported that ACSL4 and ALOX15 are crucial ferroptosis mediators by promoting the peroxidation of lipids (Qin et al. 2021; Sun et al. 2023). Some prior evidences have shown that elevating active ferrous decreased GPX4 and increased ACSL4 and ALOX15. These effects induced dysfunction of GSH and consequently oxidation of different multifunctional phospholipids (Zhang et al. 2021). In myocardial infarction, inhibition of ALOX15 was able to limit ferroptosis and lipid peroxidation while simultaneously elevating GSH levels in the PUFA-enriched cells (Ma et al. 2022). Similarly, an ALOX15 inhibitor reversed ferroptosis-associated decreased expression of the ferroptotic repressors, GPX4 and SLC7A11 in an in vitro model of neurotoxicity (Zhao et al. 2022).

In addition, the P53 gene, a powerful tumor suppressor, could provoke ferroptosis by decreasing the expression of the SLC7A11 protein, hindering the antioxidant effect (Hong et al. 2017). Furthermore, p53 promoted ALOX15 activity by inhibiting SLC7A11, thereby triggering ferroptosis in bladder cancer cells (Li et al. 2023). Previous evidences have shown that P53 is often overproduced in people with RA and animal models (Peng et al. 2022; Sun and Cheung 2002) to coincide with our results, where upregulation of P53 expression was detected in bone cells of AIA rats. In the current investigation, ticagrelor was effective in hampering ferroptotic cell death in AIA rats as revealed by the noticeable induction of the ferroptosis suppressors, FTH1, GPX4, and SLC7A11 along with the prominent diminution in the ferroptosis promoters, ACSL4 and ALOX15. This anti-ferroptotic effect could be partially ascribed to its ability to reduce P53 expression as demonstrated in AIA rats treated with ticagrelor in our study. These findings propose a potential modulatory role for ticagrelor in antioxidant defense mechanisms during AIA. In accordance with our results, the antioxidant effect of ticagrelor has been widely tackled in various investigations through its ability to boost GPX expression. Ticagrelor increased levels of GPX in rat kidneys experiencing ischemia–reperfusion injury and it was observed to attenuate ROS by elevating GPX and SOD within the renal tissue of sepsis mice model (BAĞCIOĞLU et al. 2017; Lv et al. 2022). Similar findings were observed in mice with carrageenan-induced thrombi (Zhang et al. 2022) and in both lung and heart cells of rats exposed to abdominal aorta ischemia and reperfusion (Findik et al. 2016).

Ferroptosis is strongly associated with the activation of signaling pathways driving inflammatory response. Numerous mediators, including chemokines, cytokines, and MMPs, are released in the RA synovium, triggering chronic inflammation as verified herein and previously. Fibroblast-like synoviocytes (FLSs) and chondrocytes secrete MMPs and cytokines such as TNF-α, encouraging the demolition of bone and cartilage (Ostrowska et al. 2018b). TNF-α stimulateds iron buildup in vitro by FLS from RA patients which increases the progression of RA (Chang et al. 2022). Among MMPs, MMP3 can directly break down cartilage and bone tissues (Wang et al. 2022) as well as MMP13 observed in both cartilage and synovial fluid of patients with RA, is a primary enzyme responsible for degrading the extracellular matrix, including type II collagen and proteoglycans. Moreover, MMP13 can contribute to synovial inflammation, stimulate synovial tissue overgrowth, and release cytokines from RA chondrocytes (Hu and Ecker 2021). Preceding studies demonstrated that MMP3 and MMP13 increased in chondrocytes to increase osteoarthritis progression by a ferroptosis promoter like ferric ammonium citrate (Jing et al. 2021a, 2021b). Tchetina et al. reported that by inhibiting TNF-α, iron chelators such as deferoxamine could reverse elevated MMP3 and MMP13 expression (Tchetina et al. 2016).

Several recent studies demonstrated the role of chemokines, including RANTES/CCL5, MIP-α/CCL3, and eotaxin-3/CCL26 in the progression of RA (Murayama et al. 2023; Yang et al. 2023). RANTES/CCL5 promotes the breakdown of bone and articular cartilage in RA by stimulating the production of MMP1 and MMP13 enzymes (Agere et al. 2017). A previous study reported that Met-RANTES, which blocked RANTES/CCL5, mitigated AIA in rats (Shahrara et al. 2005). Likewise, MIP-1α/CCL3 contributed to inflammatory bone loss in the AIA model by the recruitment of osteoclast precursor cells (Toh et al. 2004). Furthermore, previous research has demonstrated that MIP-1α/CCL3 either dose- or time-dependently raised MMP3 in FLS cells in a culture medium (Yang et al. 2023). Prior studies that align with our results had shown that MIP-1α/CCL3 and RANTES/CCL5 increased in AIA and CIA models, suggesting that these chemokines could be therapeutic targets in RA (Szekanecz et al. 2000; Thornton et al. 1999). From the same perspective, Chae et al. reported that eotaxin-3/CCL26 was implicated in RA as a proinflammatory mediator (Chae et al. 2005).

Besides its well-established role in lipid peroxidation during ferroptosis, ALOX15 has pro-inflammatory properties and was shown to be highly expressed in synovial RA membranes (Gheorghe et al. 2009). It was demonstrated that an ALOX15 metabolite upregulated the TNF-α expression in vascular cells and macrophages (Bolick et al. 2005; Dwarakanath et al. 2008). Furthermore, ALOX15 was involved in MMPs upregulation by TNF-α in RA synovial fibroblasts (Wu et al. 2012). In the same context, it has been reported that eotaxin-3/CCL26 was regulated by ALOX15 in the epithelial cells of the lower airways (Li et al. 2019). Similarly, ALOX15 promoted airway inflammation by inducing the expression of chemokines like RANTES/CCL5 and MIP-1α/CCL3 (Xu et al. 2021). These observations highlight the essential role of ALOX15 in promoting inflammatory response through enhancing chemokine-induced MMPs expression as well as inflammatory cells migration in RA.

Our study demonstrates that ticagrelor treatment could alleviate inflammation and promote articular repair in the AIA model. This effect was achieved by reducing TNF-α levels in bone tissue and downregulating MMP13 and MMP3 expressions, which are key enzymes included in cartilage degradation. Through the considerable downregulation of the chemokines RANTES/CCL5, MIP-1α/CCL3, and eotaxin-3/CCL26 in AlA, ticagrelor inhibited the recruitment of inflammatory cells and preserved cartilage and bone integrity, an effect that could be partially ascribed to its ability to attenuate the ALOX15-associated inflammatory response. Coherent with our results, previous studies illustrated that ticagrelor possessed an anti-inflammatory effect via various pathways. Sexton et al. proved the potency of ticagrelor to modulate inflammation by blunting TNF-α in mice that were exposed to lipopolysaccharide (Sexton et al. 2018). Furthermore, in mice model of abdominal aortic aneurysm, ticagrelor in combination with aspirin significantly reduced MMPs expression (Liu et al. 2022). Similarly, ticagrelor could reduce MMPs alone in coronary artery disease patients (Mao et al. 2019).

Furthermore, NLRP3 inflammasome is critically involved in the progression of RA and chondrocyte dysfunction by promoting the formation of pro-inflammatory cytokines contributing to cartilage and bone degradation via inducing MMPs expression (Cheng et al. 2022; Lu et al. 2023). CIA mice and AIA rats models displayed significantly elevated NLRP3 expression in comparison to the normal group (Ding et al. 2016; Zhang et al. 2016), to concur with our results. Moreover, NLRP3 level is positively related to ferroptosis in a rat model of osteoarthritis (Meihe et al. 2021). Preceding evidence proposed a potential relation between the NLRP3 inflammasome, lipid peroxidation and ferroptosis, where a buildup of ferrous iron within cells could activate the NLRP3 inflammasome (Gupta et al. 2023). In the same context, a direct proportion between NLRP3 and ACSL4 has been previously reported (Zhou et al. 2021). Kang et al. also reported that inflammasome activation was negatively regulated by GPX4 (Kang et al. 2018). Also, SLC7A11 impaired NLRP3 inflammasome expression in nonalcoholic steatohepatitis advocating the involvement of ferroptosis in NLRP3 activation (Lv et al. 2024).

Notably, this study showed that ticagrelor significantly diminished NLRP3 expression in the ankle joint of AIA rats. This effect could be attributed to ticagrelor’s capability of modulating the ferropotic markers, SLC7A11, ACSL4, and GPX4 secondary to inhibiting P53. In accordance with our results, ticagrelor demonstrated a distinct therapeutic potential by modulating the NLRP3 inflammasome in the progression of diabetic cardiomyopathy in mice (Chen et al. 2020). In a similar fashion, ticagrelor, beyond its antiplatelet effect, reduced macrophage NLRP3 in vitro in acute coronary syndrome patients (Huang et al. 2021), and following myocardial ischemia reperfusion-induced acute lung injury in rats (Dai et al. 2024). As an effort to interpret our findings concerning the crosstalk throughout the inflammatory response and ferroptosis and their relation with bone damage, the correlation between NLRP3 expression and all evaluated parameters was investigated to assess the role of ticagrelor in alleviating inflammatory burden by downregulating ferroptosis.

Compared to our findings concerning the anti-arthritic effect of ticagrelor through modulating ferroptosis and NLRP3 expression, other approved protective agents against RA have been demonstrated to target ferroptosis-associated oxidative damage and NLRP3 activation. For instance, methotrexate administration resulted in a significant downregulation of malondialdehyde and ACSL4, indicative of reduced lipid peroxidation in joint tissues of the CIA model. This effect was accompanied by upregulation of SLC7A11, GPX4, and FTH1, suggesting a potential protective mechanism against ROS and ferroptosis in RA (Zhou et al. 2023a). Moreover, it mitigated CIA inflammation by reducing NLRP3 expression (Pang et al. 2018). Additionally, hydroxychloroquine treatment, that approved for RA disease, significantly reduced NLRP3 in mouse models of renal injury (Cui et al. 2023; Tang et al. 2018). Similarly, tocilizumab, that targets the interleukin-6 receptor and is approved for RA, reduced kidney damage by preventing ferroptosis through increased levels of GPX4, xCT, and ferritin (Yang et al. 2020). In a similar fashion, recent experimental studies investigated novel protective agents against RA by exploring their ability to hinder ferroptotic cell death (Peng et al. 2022; Zhou et al. 2023a). In addition, an umbrella meta-analysis by (Bideshki et al. 2024) recently affirmed the significant efficacy of curcumin in reducing pain and improving joint function in patients with osteoarthritis which share fundamental destructive processes with RA driven by chronic inflammation and oxidative stress. This effect was previously ascribed to its ability to hinder chondrocyte ferroptosis (Zhou et al. 2023b). The demonstrated benefit of compounds targeting these shared mechanisms further supports the potential of ticagrelor's anti-inflammatory and anti-ferroptotic actions in alleviating pathological changes characteristic of inflammatory arthritis like RA.

Owing to ticagrelor's pharmacological characteristics and growing clinical use, some of its adverse effects such as bleeding tendency, dyspnea, ventricular pause, hyperuricemia and kidney damage are now getting a lot of attention in an effort to make timely changes that will guarantee the medication's safety and maximum clinical benefits (Wei et al. 2024). Notably, ticagrelor at low dosages was reported to be safe for long-term use (Cesaro et al. 2020). Consequently, the implementation of personalized treatment strategies for RA, guided by individual patient factors, remains critical for optimizing therapeutic efficacy while mitigating the risk of dose-related adverse reactions (Rauf et al. 2025; Wei et al. 2024). Moreover, prior research also highlighted hypersensitivity, including angioedema, as an unpredictable type B adverse drug reaction of anti-platelets, independent of dose, time, and frequency, and determined by host factors (Binazon et al. 2013) which is rare with ticagrelor administration (Seecheran et al. 2017).

## Conclusion

Taken all together, the present investigation focused on the anti-arthritic abilities of ticagrelor against ferroptosis-associated inflammatory response in the AIA rat model. Ticagrelor abrogated NLRP3 expression and hampered TNF-α and chemokines-induced MMPs activation to inhibit the recruitment of inflammatory cells and preserve cartilage and bone integrity. Modulating the ferroptotic inhibitors, for instance, SLC7A11, GPX4 and FTH1 besides ferroptotic promoters such as ACSL4 and ALOX15 by ticagrelor validates its anti-inflammatory and anti-ferroptotic effects in AIA. Likewise, our data revealed a strong correlation between NLRP3 suppression and modulation of P53/SLC7A11/ALOX15 signaling pathways. The findings of this study may pave the way for future clinical investigations of ticagrelor to help in the management of RA. Furthermore, it also points out the probable mechanism of ferroptosis on cartilage, which could provide further possible targets for future clinical trials for the treatment of RA.

## Limitations

Ferroptosis mechanism should be investigated in other models of arthritis. Further research is needed to explore the functional molecular mechanism of ticagrelor in treated AIA. While AIA model mimics many downstream inflammatory and destructive processes, it does not typically involve the persistent, self-perpetuating loss of self-tolerance or the robust production of specific autoantibodies seen in human RA (Jones et al. 2018). Additionally, clinical researches are required for the safety of ticagrelor. Therefore, future studies will involve detailed acute and chronic toxicity assessments, including preliminary safety assesment such as liver, kidneys, spleen, or lungs weight, and analysis of serum biomarkers for organ function ALT, AST, BUN, and creatinine to establish its full safety profile in this context. These future investigations are essential prerequisites for advancing ticagrelor towards clinical translation for RA. Additionally, our further investigations will explicitly include both male and female rats to systematically investigate sex as a biological variable in the context of ticagrelor's effects on arthritis pathogenesis. This will allow us to assess any sex-specific differences in disease progression, therapeutic response, and underlying mechanisms, fully aligning with contemporary research standards.

## Data Availability

Please contact the authors with any questions on the availability of the data.

## Abbreviations

ACSL4: Acyl-CoA synthetase long-chain family member 4
AIA: Adjuvant-induced arthritis
ALOX15: Arachidonic acid 15-lipoxygenase-1
CIA: Collagen-induced arthritis
DAB: Diaminobenzidine
DMSO: Dimethyl sulfoxide
FCA: Freund’s Complete Adjuvant
FLS: Fibroblast-like synoviocytes
FTH1: Ferritin heavy chain 1
FTL: Ferritin light chain
GPX4: Glutathione peroxidase 4
GSH: Reduced glutathione
H&E: Hematoxylin & Eosin stain
IL-18: Interleukin-18
IL-1ß: Interleukin-1β
MIP-α: Macrophage inflammatory protein-1α
MMP13: Matrix metalloproteinase 13
MMP3: Matrix metalloproteinase 3
MMPs: Matrix metalloproteinases
NLRP3: Nod-like receptor protein 3
PBS: Phosphate-buffered saline
PUFA: Peroxidase polyunsaturated fatty acids
RA: Rheumatoid arthritis
ROS: Reactive oxygen species
SLC7A11: Solute carrier family 7 member 11
TNF-α: Tumor necrotic factor alpha
